# RNA sequencing of murine mammary epithelial stem-like cells (HC11) undergoing lactogenic differentiation and its comparison with embryonic stem cells

**DOI:** 10.1186/s13104-018-3351-4

**Published:** 2018-04-11

**Authors:** Trinadha Rao Sornapudi, Rakhee Nayak, Prashanth Kumar Guthikonda, Srinivas Kethavath, Sailu Yellaboina, Sreenivasulu Kurukuti

**Affiliations:** 1 Department of Animal Biology, School of Life Sciences, University of Hyderabad, Gachibowli, Hyderabad 500046 India; 2 CR Rao Advanced Institute of Mathematics, Statistics and Computer Sciences, University of Hyderabad Campus, Hyderabad, 500046 India

**Keywords:** Mammary epithelial cells, HC11 cells, Embryonic stem cells, Transcriptome, RNA sequencing, Cellular differentiation, Glucocorticoid signaling, Prolactin signaling, Lactogenesis

## Abstract

**Objectives:**

Understanding of transcriptional networks specifying HC11 murine mammary epithelial stem cell-like cells (MEC) in comparison with embryonic stem cells (ESCs) and their rewiring, under the influence of glucocorticoids (GC) and prolactin (PRL) hormones, is critical for elucidating the mechanism of lactogenesis. In this data note, we provide RNA sequencing data from murine MECs and ESCs, MECs treated with steroid hormone alone and in combination with PRL. This data could help in understanding temporal dynamics of mRNA transcription that impact the process of lactogenesis associated with mammary gland development. Further integration of these data sets with existing datasets of cells derived from various stages of mammary gland development and different types of breast tumors, should pave the way for effective prognosis and to develop therapies for breast cancer.

**Data description:**

We have generated RNA-sequencing data representing steady-state levels of mRNAs from murine ESCs, normal MECs (N), MECs primed (P) with hydrocortisone (HC) alone and in combination with PRL hormone by using Illumina sequencing platform. We have generated ~ 58 million reads for ESCs with an average length of ~ 100 nt and an average 115 million good quality mapped reads with an average length of ~ 150 nt for different stages of MECs differentiation.

## Objective

HC11 cells are PRL responsive epithelial cell clone, derived from the COMMA1D cells and originated from the mammary gland tissue of a pregnant BALB/c mouse and are widely used model system to study the lactogenic differentiation in vitro [1]. Undifferentiated state of MECs is maintained in the presence of Insulin and epidermal growth factor (EGF). They are stimulated to differentiate by withdrawal of EGF and supplemented initially with insulin, GC and later in combination with PRL [2]. Glucocorticoids binds to cytosolic glucocorticoid receptor (GR) and functions via genomic and non-genomic pathways to accompany differential gene expression [3]. Further, PRL, a peptide hormone, upon binding to PRL receptor (PRLr) on plasma membrane initiates cascade of events which ultimately leads to the cytosolic dimerization and nuclear internalization of Stat5a/b, to promote differential expression of genes [4]. Dissecting the gene regulatory networks that act in cohort and orchestrate mammary epithelial cells differentiation under the influence of lactogenic hormones is critical for elucidating the mechanism of lactogenesis in the context of mammary gland development and differentiation. Previous studies have made an attempt to profile transcriptome of MECs during lactogenic differentiation by using microarrays [5–7], which has its inherent limitations. In this current study, we comprehensively profiled transcriptome of two independent biological replicates each for ESCs, normal, primed and PRL treated MECs by high throughput RNA sequencing method using Illumina sequencing platform. We have used these RNA-Seq datasets to derive differentially expressed genes, pathways which play key roles in orchestrating lactogenic differentiation of MECs and presented our inferences in a manuscript that is currently under review [8]. These data sets are also useful in understanding temporal dynamics of transcriptome and gene expression associated with alternative splicing specific to lactogenesis.

## Data description

We cultured R1 murine ESCs with standard recommended protocol [9] in presence of 2i medium. Normal MECs (N) were cultured in presence of insulin (5 μg/ml, Sigma # 16634) and EGF (20 ng/ml, Sigma # E4127), followed by priming (P) with HC (1 μg/ml, Sigma # H4001) and insulin (5 μg/ml, Sigma # 16634) for 48 h and in combination with PRL (5 μg/ml, NIH # NIDDK-oPRL-21) for 72 h. Total RNA was extracted from two independent replicates each for ESCs, normal, primed and PRL treated MECs by using TRIzol™ (Invitrogen # 15596026) according to manufacturer instruction. Total RNA was further purified by using G Sure cell culture RNA isolation kit (GCC Biotech # GR1003). 20μg of purified RNA from each sample was treated with 10 Units of DNAse1 (Roche # 04716728001) and were further purified by using G Sure cell culture RNA isolation kit. From each RNA sample, Ribosomal RNA was depleted by using Ribo-Zero kit (NEB#E6310L) and further mRNAs were enriched by Oligo (dT) beads. Illumina paired end library was prepared as per the NEBNext^®^ Ultra™ RNA Library Prep Kit (NEB # E7530S). All the libraries were paired end sequenced using illumina HiSeq 2500 sequencing platform. Raw sequence reads in FASTQ format were further processed to remove Illumina adaptor sequences by using Trimmomatic [10]. The resultant raw reads were compressed to .gz format and were deposited in GEO repository [11].

## Limitations

The Illumina Hi-Seq 2500 platform used in this current study, generates shorter (100–150 nt) high quality reads and annotation of full length transcript information requires overlapping sequence reads and thus requires much deep sequencing of samples. In the current datasets, we generated ~ 58, 108, 112, 127 million mappable reads for ESCs, normal (N), primed (P) and prolactin (PRL) treated MECs respectively. Though this level of sequencing depth is sufficient to derive expression and differential expression of mRNA transcripts and its alternative spliced forms, it might not be sufficient enough in finding variations to distinguish mutations, allelic or imprinting expression of genes [12]. Further, interrogation of low abundant mRNAs and long non-coding RNAs require much deeper sequencing of the samples (> 200 million). Though, one can assess few abundant microRNAs from these datasets, comprehensive characterization of miRNAs requires RNA sequencing of miRNA enriched population.

## Abbreviations

EGF: epidermal growth factor
ESC: embryonic stem cells
HC: hydrocortisone
GC: glucocorticoids
GR: glucocorticoid receptor
MECs: mammary epithelial cells
PRL: prolactin
PRLr: prolactin receptor
